# Intimate partner violence against married rural-to-urban migrant workers in eastern China: prevalence, patterns, and associated factors

**DOI:** 10.1186/s12889-016-3896-x

**Published:** 2016-12-07

**Authors:** Li Chen, Zonghuo Yu, Xianming Luo, Zhaoxin Huang

**Affiliations:** 1 Department of Psychology, School of Psychiatry, Wenzhou Medical University, Wenzhou, China; 2 School of Psychology, Jiangxi Normal University, Nanchang, China; 3 Department of Humanities and Social Sciences, Wenzhou Medical University, Wenzhou, China

**Keywords:** Intimate partner violence, Married rural-to-urban migrant workers, Prevalence, Patterns, Factors

## Abstract

**Background:**

Intimate partner violence (IPV) is a significant public health issue among married rural-to-urban migrant workers, the largest group of internal migrants in China. This study aims to explore the prevalence, patterns and associated factors of intimate partner violence against married rural-to-urban migrant workers in eastern China.

**Methods:**

A cross-sectional study was conducted in Zhejiang province in China between July 2015 and April 2016, and a total of 1,744 married rural-to-urban migrant workers ultimately took part in the study. Conflict Tactics Scales and several short demographic questions were applied. Data were principally analyzed with logistic regression.

**Results:**

The majority of married rural-to-urban migrant workers were middle-aged couples with a low education level and a relatively long-term duration of migration in fixed migrant cities. Nearly 45% of married rural-to-urban migrant workers were experienced at least one incident of intimate partner violence during the past 12 months. The joint occurrence of multiple forms of violence is the most commonly reported features of intimate partner violence, especially three overlapping patterns of intimate partner violence. Some individual (education and age), relationship (marital satisfaction, premarital sex and extramarital affairs) and social (duration of migration and number of migratory cities) factors of the respondents, were negatively or positively associated with intimate partner violence against married rural-to-urban migrant workers.

**Conclusion:**

The results indicated that one out of two married rural-to-urban migrant workers experienced at least one incident of intimate partner violence during the past 12 months in China. Accordingly, there is an obvious demand of intervention and treatment activities to prevent and reduce the occurrence of intimate partner violence among the millions of migrant workers in China.

**Electronic supplementary material:**

The online version of this article (doi:10.1186/s12889-016-3896-x) contains supplementary material, which is available to authorized users.

## Background

Intimate partner violence (IPV) is a serious and widespread problem worldwide. It is a domestic violence by a spouse or partner in an intimate relationship against the other spouse or partner [1], and the violence may be mutual, in which case the relationship may be described as a violent relationship [2]. The dominant forms of IPV included physical, sexual and psychological abuse [3]. Furthermore, Intimate partner violence could occur among heterosexual or same-sex couples, and does not require sexual intimacy [4].

IPV has been shown to be the most common type of violence against married couples, especially among women groups. For example, a study, based on 48 population-based surveys from developing and developed countries and conducted by World Health Organization (WHO) in 2002, found that 13–61% of women in countries across the world reported having experienced IPV from their partners [5]. Another national intimate partner and sexual violence survey in United States found that more than one in three women (35.6%) and more than 1 in 4 men (28.5%) have experienced rape, physical violence, and/or stalking by an intimate partner in their lifetime [6]. In Africa, almost seven in ten women had previously been abused by their partners and the overlap of psychological, physical, and sexual violence was 57% [7]. In spite of the definitions and methodological differences, several population-based studies indicated that the prevalence rate of IPV in general population is approximately 10–71% [5, 6, 8, 9].

Meanwhile, IPV affects physical health and mental health of IPV survivors and their families adversely through direct pathways, such as injury, humiliation and isolation, and indirect pathways, such as family function, attachment, marital satisfaction and other factors [10–13]. A history of experiencing violence is therefore a risk factor for many diseases and conditions. Current research indicates that the more severe the abuse, the greater its impact on a victim’s physical and mental health, and this impact of IPV could last a lifetime and span generations [14, 15]. Furthermore, the impact over time of different types and multiple episodes of abuse appears to be cumulative [16].

These research not only have significantly advanced current knowledge concerning IPV among different groups, but also have motivated new important research questions. For instance, rural-to-urban migrant workers in china are now experiencing the largest mass migration of people from the countryside to the city in history [17]. Migration is an extremely stressful experience since being away from home is associated with an increase in risk behaviors including all forms of violence. Furthermore, migrant workers often have to move from place to place with constant changes in job or living conditions, and some of them even have to be separated from their spouses for long periods of time [18, 19]. Is it possible for rural-to-urban migrant workers to have higher incidence and level of IPV compared with the general population? What are the risk and protective factors associated with IPV among married rural-to-urban migrant workers in China?

The present study, based on those existing studies, aims to further explore IPV among married rural-to-urban migrant workers with making the following three specific efforts.

First, we have chosen married rural-to-urban migrant workers as our subjects. In China, rural-to-urban migrant workers are a special group formed by Chinese peasants in the process of social transformation during the course of urbanization. They refer to farmers who move from rural to urban areas within China in pursuit of employment and better living standards, without no permanent urban residency (“hukou”). According to the National Bureau of Statistics, it was estimated that there were 277 million rural-to-urban migrant workers in 2015, accounting for about 20% of the total population. Among them, an estimated 203 million were married [20]. Although rural-to-urban migrant workers make up half of urban workforce and account for half of the country’s GDP in China, most of them have to face negative conditions including high pressure from work, poverty, low social status, unsure self-identification, wandering life and so on [18, 19, 21, 22]. Furthermore, many of married rural-to-urban migrant workers have to be separated from their spouses for long periods of time. These conditions have been demonstrated in many research to be the known risk factors of IPV [3, 7, 14].

Second, we focus on the prevalence and pattern of IPV among married rural-to-urban migrant workers. In China, the issue of IPV has received limited attention until the Fourth World Conference on Women (1995) was hosted in Beijing. In the past two decades, the relevant studies on IPV in China have mainly been conducted on theoretical mechanism of IPV. The first empirical and wide-scale study on IPV in china was carried out in 2000. It was found that 34.7% of 3,543 investigated women admitted that they had experienced physical, psychological or sexual violence [23]. The following investigations and research have focused largely on urban residences, farmers and female group. Tiwari A and her colleagues, for instance, surveyed 3,245 pregnant women in Hong Kong to examine patterns and risk factors of IPV against pregnant women. They found that about 9% of the pregnant women reported having been abused by their partners in the preceding year [24]. Another survey study with 1,577 women in a rural county in western China found that the lifetime prevalence of physical assault, psychological aggression, and sexual coercion was 16.3, 30, and 1.8% among the investigated subjects, and physical abused victims were at over four times greater risk of having suicidal intention than those who had not suffered physical assault [25]. However, studies on IPV against married migrant workers are limited to only one or two types of violence and do not reveal the overall prevalence of IPV among married migrant workers, especially in the context of Chinese society.

Third, according to the ecological model theory of violence, the causes of IPV are diverse and there is no single factor that explains further why some individuals are violent. Rather, violence is a result of individual, relationship and societal factors [26]. Therefore, the present study would explore the risk and protective factors for IPV against married migrant workers at three levels: individual, relationship and societal level. First, individual-level factors include biological and personal history factors that may increase or decrease the likelihood that an individual will become a victim or perpetrator of IPV. Second, relationship-level factors were mainly concentrated on the increase or decrease risks as a result of material relations with spouse. Third, societal-level factors in this study focus on the migrating experience such as duration of migration, number of migratory cities and other migrating factors that are associated with people becoming victims or perpetrators of IPV.

The aims of the present paper were to (1) examine prevalence and pattern of IPV among married rural-to-urban migrant workers in Eastern China and (2) identify the risk and protective factors for IPV at three levels: individual, relationship and societal level.

## Methods

### Participants

In this study, rural-to-urban migrant workers were defined as those who were 18 years of age or older, possess a legal rural hukou (“户口”, formally registered permanent residents of in a rural area in China), and have been granted the legal right to work temporarily in urban and prosperous coastal regions for at least six months [27]. Migrant workers could be divided into “first-generation” and “second-generation or new generation” migrant workers [18]. The first-generation migrant workers refer to those migrant workers born before 1980, who began to flow from the rural situation into the migrant situation in the city in the 1980s and 1990s [18]. The second-generation migrant workers refer to those who were born after 1980s with their registered permanent residence being in the countryside, but they have been coming to work in the city in 1990s [18, 28].

A cross-sectional study was conducted in Zhejiang province in China between July 2015 and April 2016, and a total of 1,744 married rural-to-urban migrant workers ultimately took part in the study. The final eligible participants were identified by the multistage probability sampling method as follows.

In stage 1, four cities, Hangzhou, Ningbo, Wenzhou and Jinghua, were selected from Zhejiang Province in China, respectively. We chose these four cities of Zhejiang Province as our study cites since Zhejiang was one of the most economically developed provinces in China’s four economical zones with a migrant population of estimated 14 million in 2015, accounting for around one-thirds of the province’s population. Meanwhile, more than two-thirds of rural-to-urban migrant workers in this area are currently in Hangzhou, Ningbo, Wenzhou and Jinghua. In stage 2, three districts in each of the four study cities were randomly selected, and these districts represented the inner-city, suburban and urban fringe zone. In stage 3, two residential sub-districts with a high density of rural–urban migrants in each of the three studies districts were randomly selected. In stage 4, a quota-sampling procedure based on six occupational clusters was applied to ensure that the sample was representative of the migrant worker population in Zhejiang. According to the figures released by the National Bureau of Statistics of China in 2015, in Zhejiang, the proportions of migrant workers in six occupational clusters, manufacturing, wholesale and retail, construction, domestic service, transportation, and hotels and restaurants, were approximately 45, 13, 11, 9, 6, and 4%, respectively [20]. Worksites in these six clusters were then used as the sampling units and a total of 176 worksites were selected from two sub-districts according to the occupational cluster and then 20% of these worksites were randomly sampled by type. In stage 5, four criteria have been used to select eligible participants from the sampling units: (1) A rural-to-urban migrant worker was defined as an individual who was registered at a rural residence, had been working in a urban region for at least 6 months without obtaining permanent residence, and was aged 18 or above. (2) An eligible participant had to be married at least one year; (2) An eligible participant could speak or read Chinese characters; (3) An eligible participant was willing to participate in the study and to sign the informed consent.

Hence, 1,864 married rural-to-urban migrant workers were eligible for the study and consent with the study procedures, and then1,744 made valid replies, yielding a response rate of 93.56%. Details of socio-demographic characteristics of the sample are displayed in Table 1 (Additional file 1: Data used in this paper).

**Table 1:** Socio-demographic characteristics of the participants (*N* = 1,744)

Variables		*Mean*	*SD*	*n*	%
Gender	Men			873	50.1
	Women			871	49.9
Age		37.74	9.01		
Age group	≤37 years (second generation)			800	45.9
	>37 years (first generation)			944	54.1
Education (years)		9.29	2.62		
Education group	Primary school or lower			147	8.4
	Junior high school			946	54.2
	Senior high school			414	23.7
	College			237	13.6
Region of origin	North			583	33.4
	South			1161	66.6
Monthly income (RMB: yuan)		3882.96	2022.12		
	≤ 2500			179	10.3
	2501–3500			846	48.5
	>3500			719	41.2
Duration of migration (years)		12.66	6.89		
	1–6			364	20.9
	7–12			539	30.9
	13–20			631	36.2
	≥21			210	12.0
Number of migratory cities	1–2			1054	60.5
	3–4			550	31.5
	≥5			140	8.1
Marital status	Frist marriage			1633	93.6
	Remarriage			111	6.4
Lifestyle with your spouse	Living together			309	17.7
	Living in separate places			1425	81.7
	Missing			10	0.5
Marital satisfaction	Satisfied			1384	79.4
	Ok			321	18.4
	Dissatisfied			39	2.2
Premarital sex	No			1171	67.1
	Yes			572	32.8
	Missing			1	-
Marriage derailment	No			1601	91.1
	Yes			138	7.9
	Missing			5	0.3

### Procedure

The present study consisted of the following two-steps procedure. First, in the pilot study, the pre-test was conducted with a convenience sample of 50 married rural-to-urban migrant workers from the target population to evaluate clarity, comprehensiveness, and acceptability of questionnaires. Some amendments were made prior to the initial delivering. Secondly, in the formal study, after fully understanding the purpose and the procedure of the study, the eligible participants signed the informed consent. With their agreement, one envelope with the questionnaire and instructions was handed to every participant. The instructions told them how to fill in the questionnaire. Participants filled out the 20-minute questionnaires in quiet and comfortable reading rooms in factory (one to six participants at one time), then placed the completed questionnaire inside an envelope and gave it back to the researchers. Third, at the end of the survey, each of participants received a leaflet detailing local support services or network counseling service for domestic violence, sexual abuse and other mental health problems. Fourth, upon completion, everyone was given a towel, a tooth brush and a tooth paste as a token of appreciation. The questionnaires were administered by trained researchers, including faculty members and postgraduate students from Wenzhou Medical University, who had been provided systematic training before formal study. The questionnaires were anonymous and all participants took part in the study voluntarily.

This study was done in compliance with the Helsinki Declaration, and was reviewed and approved by the Ethics Committee of Wenzhou Medical University.

### Measures

#### Socio-demographics

A socio-demographic questionnaire consisted of three parts. The first part is the individual-level information including age, gender, education, region of origin (north vs. south), monthly income and other basic demographic information. The second part is the relationship-level information. In this study, relationship-level information focuses on marital information including marital status, lifestyle with your spouse (living together vs. living in separate places), marital satisfaction, premarital sex (yes vs. no) and extramarital affairs (yes vs. no). The third part is societal-level information. In this study, societal-level information focuses on migratory information including duration of migration and number of migratory cities.

#### Intimate partner violence

Intimate partner violence was measured by the Chinese version of short form of the revised Conflict Tactics Scales (CTS2) [29, 30] (Additional file 2: A transcript of the questionnaire). The short form CTS2 scales is a 20-item self-reported instrument for measuring the extent to which intimate partners’ self-report abuse from or toward one another and also the prevalence and frequency of this violence. Items are combined to form five subscales: negotiation, psychological aggression, physical assault, sexual coercion and injury. Each of these 20 items is rated on a 7-points Likert scale, ranging from 0 (never) to 6 (more than 20 times in the past year). For the Chinese version, the factor structure, good reliability and validity were demonstrated [30].

In the present study, intimate partner violence is defined as self-reported physical, sexual and emotional violence victimization by a current or former spouse, and it was assessed by prevalence and frequency of three forms of violence (psychological IPV, physical IPV and sexual IPV). Firstly, psychological IPV was assessed by psychological aggression subscale of the Chinese version of short form CTS2. Specifically, the self-reported frequency of psychological abuse from the other spouse in the past year was recorded. Examples of psychological IPV include: insulted, swore, shouted, yelled by spouse and made me feel bad about myself; destroyed belongings or threatens to hit. The psychological IPV subscale consists of four items with high degree of internal consistency reliability among the Chinese participants (Cronbach’s α = 0.72) [30].

Secondly, physical IPV was assessed by physical assault subscale of the Chinese version of short form CTS2. Specifically, the self-reported frequency of physical assault from the other spouse in the past year was recorded. Examples of physical IPV include: pushed, shoved, slapped, punched, kicked and beatin of the spouse. The physical assault subscale consists of four items with good degree of internal consistency reliability among the Chinese participants (Cronbach’s α = 0.88) [30].

Thirdly, sexual IPV was assessed by sexual coercion subscale of the Chinese version of short form CTS2. Specifically, the self-reported frequency of intimate partner’s sexual coercion from the other spouse in the past year was recorded. Examples of sexual IPV include: physically forced to have sex; did not want to have sex or forced to have sex without a condom. The sexual coercion subscale consists of four items with good degree of internal consistency reliability among the Chinese participants (Cronbach’s α = 0.93) [30].

### Data analysis

First of all, socio-demographic characteristics of the sample were described by the number and the percentage of each category for categorical variables. Secondly, we calculated prevalence estimates, frequency and overlap in different forms of IPV among married migrant workers in the past 12 months. Thirdly, multivariate binary logistic regression analysis was used to determine the risk factors for IPV of married migrant workers, and crude odds ratios and adjusted odds ratios (OR) and 95% confidence intervals (CIs) for OR were calculated. In logistic regression modeling, overall IPV, psychological IPV, physical IPV, and sexual IPV were considered as the dependent variables respectively, and the demographic characteristics of the married migrant workers including individual level determinants (age, gender, education, region of origin, income), marital determinants (marital status, lifestyle with spouse, marital satisfaction, premarital sex and extramarital affairs) and migratory determinants (duration of migration and number of migratory cities) were considered as the independent variables. All statistical analyses were performed with the use of SPSS statistics package (version 18.0) and all reported *P*-values are 2-tailed with statistical significance set at 0.05.

## Results

### Socio-demographic characteristics

Of the 1,744 participants, 875 (50.1%) were male and 871 (49.9%) were female. The participants’ age ranged from 19 to 61 years old (37.74 ± 9.01 years). 45.9% of migrant workers could be classified as “second-generation” migrants. Their duration of marriage ranged from 1 to 45 years (13.15 ± 9.70 years). Over a half of the married migrant workers only had a junior high school education or below, and the average time in schooling was 9.29 ± 2.62 years. Regarding monthly income, the average monthly income of the participants was 3883 RMB (approximately US$596). Only 10.3% of the participants earned less than 2500 Yuan (RMB,100RMB ≈ 15USD), nearly half (48.5%) earned 2501–3500 Yuan (RMB), and 41.2% earned more than 3500 Yuan (RMB). The average duration of migration was 12.66 ± 6.89 years, and almost 60.5% of the participants reported having migrated to only one or two cities, while 8.1% reported having been migrated to more than five cities. In terms of marital status, 1633 participants (93.6%) were first married and 111 were remarried (6.4%). The majority of participants were satisfied with their marriage (79.4%) and living with their spouse together every day (78.2%). It is worth mentioning that nearly 32.8% of the participants reported having sexual intercourse before marriage and 7.9% reported having an affair after marriage. The general demographic, migration and marital characteristics of the married migrant workers are summarized in Table 1. It demonstrates that the majority of the married migrant workers were middle-aged couples with a low education level and a relatively long-term duration of migration in fixed migrant cities.

### Prevalence and pattern of IPV among married rural-to-urban migrant workers

Table 2 shows prevalence and frequency of different forms of IPV against married rural-to-urban migrant workers in the sample. In general, out of the 1,744 married migrant workers, almost a half (44.2%, 95% CI: 41.8–46.5%) experienced at least one act of physical, psychological or sexual IPV during the past 12 months. Specifically, over one thirds of the respondents (39.0%, 95% CI: 36.8–41.3%) experienced one episode of psychological violence in the past 12 months, followed by 19.5% of them experienced physical IPV (19.04%, 95% CI: 17.2–20.9%), and the percentage of sexual IPV was lowest, only 19.27% (16.06%, 95% CI: 14.3–17.8%). Meanwhile, three forms of IPV appear to show similar characteristics on the frequency distributions, in which participants having history of any form of IPV mostly experienced two and five times in the past 12 months, and once or over five times were minors.

**Table 2:** Past 12 months prevalence and frequency of intimate partner violence among the married migrant workers

Forms of violence	Prevalence in the past 12 months		Frequency in the past 12 months		
	Number %	95% CI	Once	2-5times	>5 times
Psychological IPV					
Insulted/swore/shouted/yelled me that made feel bad about myself	622 (35.67)	(0.33–0.38)	229 (13.13)	300 (17.20)	93 (5.33)
destroyed something belonging to me or threatened to hit me	294 (16.86)	(0.15–0.19)	126 (7.22)	121 (6.94)	47 (2.69)
At least one episode of psychological IPV	681 (39.05)	(0.37–0.41)	206 (11.81)	326 (18.69)	149 (8.54)
Physical IPV					
Pushed /shoved/slapped me	276 (15.83)	(0.14–0.18)	100 (5.73)	115 (6.59)	61 (5.19)
punched /kicked / beat-me-up	272 (15.59)	(0.14–0.17)	108 (6.19)	106 (6.08)	58 (3.32)
At least one episode of Physical IPV	332 (19.04)	(0.17–0.21)	67 (3.84)	155 (8.89)	100 (5.73)
Sexual IPV					
Physically forced to have sex	252 (14.45)	(0.13–0.16)	94 (5.39)	94 (5.34)	64 (3.67)
Did not want to have sex or forced to sex without a condom	230 (13.19)	(0.12–0.15)	76 (4.36)	92 (5.28)	62 (3.56)
At least one episode of Sexual IPV	280 (16.06)	(0.14–0.18)	37 (2.12)	123 (7.05)	120 (6.88)
At least one of the three violence	770 (44.15)	(0.42–0.47)			

**Fig. 1:**
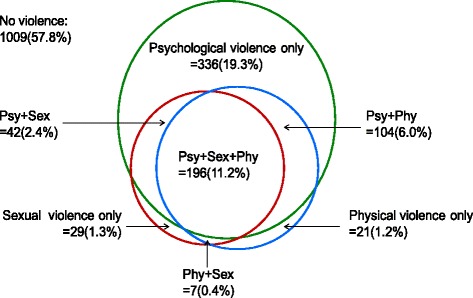
Overlaps between difference forms of intimate partner violence

The different forms of IPV and their overlapping are shown in the Venn diagrams in Fig. 1. It illustrates that the most commonly occurring form of violence was psychological IPV alone (19.3%) compared to physical IPV alone (1.2%) and sexual IPV alone (1.3%). However, among these married migrant workers experiencing IPV, over half of them experienced multiple forms of violence from their intimate partners in the past 12 months, especially three overlapping patterns of IPV (11.2%).

### Factors associated with IPV

#### Factors associated with overall IPV

In the regression model of overall IPV, as shown in Table 3, the associations between overall IPV and the demographic characteristics of married migrant workers were explored. The second-generation married migrant workers were (AOR 0.75, 95% CI: 0.58–0.96) less likely to report IPV than the first-generation married migrant workers. Furthermore, compared to those married migrant workers with primary school education level or lower, married migrant workers with junior high school (AOR 0.66, 95% CI:0.47–0.96) and senior high school (AOR 0.58, 95% CI: 0.39–0.88) level were less likely to report IPV. Meanwhile, it is worthwhile to note that respondents who were dissatisfied with their marriage (AOR 5.59, 95% CI: 2.25–13.87) or somewhat satisfied with their marriage (AOR 1.71, 95% CI: 1.32–2.22) were more likely to report current experiences of IPV than those satisfied with their marriage. In the same way, for a married migrant worker who had sexual intercourse before marriage were more likely to have experienced IPV after marriage (AOR 1.51, 95% CI:1.21–1.89) than those without premarital sex. Also, married migrant workers who have affairs after marriage were nearly at twice as high of a risk to experience IPV than those without having affairs (AOR 1.84, 95% CI: 1.26–2.71). More results are presented in Table 3.

**Table 3:** Factors associated with intimate partner violence against married migrant workers

	Intimate partner violence		cOR (95% CI)	*p*	aOR (95% CI)	*p*
	Yes (*n* = 770)	No (*n* = 974)				
	*n* (%)	*n* (%)				
Gender						
Men	379 (49.2)	494 (50.7)	1.00 (reference)		1.00 (reference)	
Women	391 (50.8)	480 (49.3)	1.06 (0.88–1.28)	0.53	1.04 (0.84–1.28)	0.74
Age group						
>37 years(first generation)	382 (49.6)	418 (42.9)	1.00 (reference)		1.00 (reference)	
≤37 years (second generation)	388 (50.4)	556 (57.1)	0.71 (0.58–0.85)	0.00	0.75 (0.58–0.96)	0.02
Education group						
Primary school or lower	78 (10.1)	69 (7.2)	1.00 (reference)		1.00 (reference)	
Junior high school	408 (53.0)	538 (55.2)	0.67 (0.47–0.95)	0.03	0.66 (0.46–0.96)	0.03
Senior high school	177 (23.0)	237 (24.3)	0.66 (0.45–0.96)	0.03	0.58 (0.39–0.88)	0.01
College	107 (13.9)	130 (13.3)	0.73 (0.48–1.10)	0.13	0.64 (0.40–1.02)	0.06
Region of origin						
North	246 (31.9)	337 (34.6)	1.00 (reference)		1.00 (reference)	
South	524 (68.1)	637 (65.4)	1.13 (0.92–1.38)	0.24	1.24 (0.98–1.54)	0.05
Monthly income (RMB: yuan)						
≤2500	77 (10.0)	102 (10.5)	1.00 (reference)		1.00 (reference)	
2501–3500	370 (48.1)	476 (48.9)	1.03 (0.74–1.43)	0.86	1.08 (0.77–1.52)	0.66
>3500	323 (41.9)	396 (40.7)	1.08 (0.78–1.50)	0.65	1.07 (0.76–1.55)	0.66
Duration of migration (years)						
1–6	171 (22.2)	193 (19.8)	1.00 (reference)		1.00 (reference)	
7–12	256 (33.2)	283 (29.1)	1.02 (0.78–1.33)	0.88	0.55 (0.82–1.46)	1.10
13–20	250 (32.5)	381 (39.1)	0.74 (0.57–0.96)	0.02	0.44 (0.65–1.21)	0.89
≥21	93 (12.1)	117 (12.0)	0.89 (0.64–1.26)	0.53	0.47 (0.77–1.76)	1.16
Number of migratory cities						
1–2	446 (57.9)	608 (62.4)	1.00 (reference)		1.00 (reference)	
3–4	260 (33.8)	290 (29.8)	1.22 (0.99–1.50)	0.06	1.17 (0.94–1.45)	0.17
≥5	64 (8.3)	76 (7.8)	1.15 (0.81–1.64)	0.45	0.87 (0.59–1.28)	0.48
Marital status						
First marriage	665 (86.4)	869 (89.2)	1.00 (reference)		1.00 (reference)	
Remarriage	105 (13.6)	95 (10.8)	1.79 (1.21–2.64)	0.00	1.34 (0.86–2.08)	0.19
Lifestyle with your spouse						
Living together	155 (20.3)	154 (15.9)	1.00 (reference)		1.00 (reference)	
Living in separate places	609 (79.7)	816 (84.1)	1.35 (1.05–1.73)	0.02	1.03 (0.79–1.34)	0.85
Marital satisfaction						
Satisfied	563 (73.1)	821 (84.3)	1.00 (reference)		1.00 (reference)	
Ok	175 (22.7)	146 (15.0)	1.75 (1.37–2.23)	0.00	1.71 (1.32–2.22)	0.00
Dissatisfied	32 (4.2)	7 (0.7)	6.67 (2.92–15.21)	0.00	5.59 (2.25–13.89)	0.00
Premarital sex						
No	467 (60.7)	704 (72.3)	1.00 (reference)		1.00 (reference)	
Yes	302 (39.3)	270 (27.7)	1.69 (1.38–2.06)	0.00	1.51 (1.21–1.89)	0.00
Extramarital affairs						
No	677 (11.5)	924 (94.9)	1.00 (reference)		1.00 (reference)	
Yes	88 (11.5)	50 (5.1)	2.40 (1.67–3.45)	0.00	1.84 (1.26–2.71)	0.00

#### Factors associated with psychological IPV

In the regression model of psychological IPV, as shown in Table 4, the second-generation married migrant workers were (AOR 0.72, 95% CI:0.56–0.93) less likely to report IPV than the first-generation married migrant workers. On the other hand, marital dissatisfaction (AOR 5.06, 95% CI: 2.22–11.54), premarital sex (AOR 0.72, 95% CI: 1.12–1.75) and infidelity after marriage (AOR 1.76, 95% CI: 1.21–2.56) increased the odds of exposure to psychological IPV in married migrant workers.

**Table 4:** Factors associated with Psychological IPV against married migrant workers

	Psychological violence		cOR (95% CI)	*p*	aOR (95% CI)	*p*
	Yes (*n* = 676)	No (*n* = 1054)				
	n (%)	n (%)				
Gender						
Men	329 (48.3)	544 (51.2)	1.00 (reference)		1.00 (reference)	
Women	352 (51.7)	519 (48.8)	1.12 (0.94–1.36)	0.24	1.08 (0.86–1.34)	0.47
Age group						
>37 years(first generation)	324 (47.6)	605 (56.9)	1.00 (reference)		1.00 (reference)	
≤37 years (second generation)	357 (52.4)	458 (43.1)	0.69 (0.57–0.83)	0.00	0.72 (0.56–0.93)	0.01
Education group						
Primary school or lower	68 (10.0)	79 (7.4)	1.00 (reference)		1.00 (reference)	
Junior high school	360 (52.9)	586 (55.1)	0.71 (0.50–1.01)	0.06	0.71 (0.49–1.03)	0.07
Senior high school	161 (23.6)	253 (23.8)	0.74 (0.57–1.08)	0.12	0.67 (0.45–1.01)	0.06
College	92 (13.5)	145 (13.6)	0.74 (0.49–1.12)	0.15	0.64 (0.40–1.02)	0.06
Region of origin						
North	222 (32.6)	361 (34.0)	1.00 (reference)		1.00 (reference)	
South	459 (67.4)	702 (66.0)	1.06 (0.87–1.30)	0.56	1.16 (0.94–1.44)	0.18
Monthly income (RMB: yuan)						
≤2500	67 (9.8)	112 (10.5)	1.00 (reference)		1.00 (reference)	
2501–3500	326 (47.9)	520 (48.9)	1.05 (0.75–1.46)	0.78	1.10 (0.78–1.56)	0.58
>3500	288 (42.3)	431 (40.5)	1.12 (0.80–1.56)	0.52	1.14 (0.80–1.65)	0.47
Duration of migration (years)						
1–6	148 (21.7)	216 (20.3)	1.00 (reference)		1.00 (reference)	
7–12	233 (34.2)	306 (28.8)	1.11 (0.85–1.46)	0.44	1.18 (0.89–1.58)	0.25
13–20	214 (31.4)	417 (39.2)	0.75 (0.57–0.98)	0.03	0.90 (0.66–1.23)	0.51
≥21	86 (12.6)	124 (11.7)	1.01 (0.72–1.43)	0.95	1.35 (0.87–2.01)	0.19
Number of migratory cities						
1–2	400 (58.7)	654 (61.5)	1.00 (reference)		1.00 (reference)	
3–4	226 (33.2)	324 (30.5)	1.14 (0.92–1.41)	0.22	0.45 (1.09–0.88)	0.24
≥5	55 (8.1)	85 (8.0)	1.06 (0.74–1.52)	0.76	0.24 (0.79–0.53)	0.12
Marital status						
First marriage	628 (92.2)	1005 (94.5)	1.00 (reference)		1.00 (reference)	
Remarriage	53 (7.8)	58 (5.5)	1.46 (1.00–2.15)	0.05	1.20 (0.77–1.86)	0.42
Lifestyle with your spouse						
Living together	546 (80.5)	879 (83.2)	1.00 (reference)		1.00 (reference)	
Living in separate places	132 (19.5)	177 (16.8)	1.20 (0.94–1.54)	0.15	0.92 (0.70–1.21)	0.54
Marital satisfaction						
Satisfied	499 (73.3)	885 (83.3)	1.00 (reference)		1.00 (reference)	
Ok	153 (22.5)	168 (15.8)	1.62 (1.26–2.06)	0.00	1.60 (1.24–2.07)	0.00
Dissatisfied	29 (4.3)	10 (0.9)	5.14 (2.49–10.64)	0.00	5.06 (2.22–11.54)	0.01
Premarital sex						
No	416 (61.2)	755 (71.0)	1.00 (reference)		1.00 (reference)	
Yes	264 (38.8)	308 (29.0)	1.56 (1.27–1.91)	0.00	1.40 (1.12–1.75)	0.00
Marriage derailment						
No	602 (88.7)	999 (94.2)	1.00 (reference)		1.00 (reference)	
Yes	77 (11.3)	61 (5.8)	2.10 (1.48–2.98)	0.00	1.76 (1.21–2.56)	0.00

#### Factors associated with physical IPV

In the regression model of physical IPV, as shown in Table 5, the protective effects of education and migration duration of married migrant workers were found to be significant in the current experiences of physical IPV. For example, respondents with junior high school (AOR 0.62, 95% CI: 0.41–0.96), senior high school (AOR 0.50, 95% CI: 0.31–0.82) and college education (AOR 0.52, 95% CI: 0.30–0.91) were less likely to report physical IPV than those with primary school or lower. Similarly, respondents with migration duration of 7–12 years (AOR 0.62, 95% CI: 0.44–0.88), migration duration of 13–20 years (AOR 0.58, 95% CI: 0.40–0.86) and migration duration of over 21 years (AOR 0.56, 95% CI: 0.33–0.97) were less likely to report physical IPV than those with migration duration of 1–6 years. Meanwhile, some other socio-demographic factors were also identified as significant risk factors associated with physical IPV among married migrant workers. Compared to respondents migrating to one or two cities, those migrating above five cities were about twice (AOR 1.73, 95% CI: 1.11–2.70) more likely to report physical IPV. Furthermore, marital dissatisfaction (AOR 3.89, 95% CI: 1.87–8.08), premarital sex (AOR 1.44, 95% CI: 1.10–1.90) and infidelity after marriage (AOR 2.12, 95% CI: 1.41–3.18) were statistically significant risk factors for physical IPV.

**Table 5:** Factors associated with Physical IPV against married migrant workers

	Physical IPV		cOR (95% CI)	*p*	aOR (95% CI)	*p*
	Yes (*n* = 332)	No (*n* = 1408)				
	n (%)	n (%)				
Gender						
Men	160 (48.2)	712 (50.6)	1.00 (reference)		1.00 (reference)	
Women	172 (51.8)	696 (49.4)	1.10 (0.87–1.40)	0.44	1.11 (0.85–1.45)	0.45
Age group						
>37 years(first generation)	148 (44.6)	778 (55.3)	1.00 (reference)		1.00 (reference)	
≤37 years (second generation)	184 (55.4)	630 (44.7)	0.65 (0.51–0.83)	0.00	0.81 (0.59–1.11)	0.18
Education group						
Primary school or lower	38 (11.4)	109 (7.7)	1.00 (reference)		1.00 (reference)	
Junior high school	174 (52.4)	768 (54.5)	0.65 (0.43–0.97)	0.04	0.62 (0.41–0.96)	0.03
Senior high school	73 (22.0)	341 (24.2)	0.61 (0.39–0.96)	0.03	0.50 (0.31–0.82)	0.01
College	47 (14.2)	190 (13.5)	0.71 (0.44–1.16)	0.17	0.52 (0.30–0.91)	0.02
Region of origin						
North	103 (31.0)	480 (34.1)	1.00 (reference)		1.00 (reference)	
South	229 (69.0)	928 (65.9)	1.15 (0.89–1.49)	0.29	1.30 (0.99–1.70)	0.06
Monthly income (RMB: yuan)						
≤2500	23 (6.9)	156 (11.1)	1.00 (reference)		1.00 (reference)	
2501–3500	165 (49.7)	678 (48.2)	1.04 (0.76–1.45)	0.80	1.09 (0.79–1.54)	0.67
>3500	144 (43.4)	574 (40.8)	1.09 (0.78–1.52)	0.58	1.11 (0.81–1.59)	0.59
Duration of migration (years)						
1–6	92 (27.7)	272 (19.3)	1.00 (reference)		1.00 (reference)	
7–12	103 (31.0)	436 (31.0)	0.70 (0.51–0.96)	0.03	0.62 (0.44–0.88)	0.01
13–20	104 (31.3)	524 (37.2)	0.59 (0.43–0.81)	0.00	0.58 (0.40–0.86)	0.01
≥21	33 (9.9)	176 (12.5)	0.55 (0.36–0.86)	0.01	0.56 (0.33–0.96)	0.03
Number of migratory cities						
1–2	179 (53.9)	872 (61.9)	1.00 (reference)		1.00 (reference)	
3–4	112 (33.7)	437 (31.0)	1.25 (0.96–1.62)	0.10	1.20 (0.91–1.58)	0.21
≥5	41 (12.3)	99 (7.0)	2.02 (1.36–3.00)	0.00	1.73 (1.11–2.70)	0.02
Marital status						
First marriage	299 (90.1)	1330 (94.5)	1.00 (reference)		1.00 (reference)	
Remarriage	33 (9.9)	78 (5.5)	1.88 (1.23–2.88)	0.00	1.41 (0.86–2.33)	0.18
Lifestyle with your spouse						
Living together	255 (77.5)	1166 (83.2)	1.00 (reference)		1.00 (reference)	
Living in separate places	74 (22.5)	235 (16.8)	1.44 (1.07–1.93)	0.02	1.01 (0.73–1.39)	0.96
Marital satisfaction						
Satisfied	223 (67.2)	1158 (82.2)	1.00 (reference)		1.00 (reference)	
Ok	90 (27.1)	230 (16.3)	2.03 (1.53–2.70)	0.00	1.88 (1.39–2.55)	0.00
Dissatisfied	19 (5.7)	20 (1.4)	4.93 (2.59–9.39)	0.00	3.88 (1.87–8.07)	0.00
Premarital sex						
No	187 (56.3)	981 (69.7)	1.00 (reference)		1.00 (reference)	
Yes	145 (43.7)	426 (30.3)	1.79 (1.40–2.28)	0.00	1.44 (1.10–1.90)	0.01
Marriage derailment						
No	278 (84.0)	1319 (93.9)	1.00 (reference)		1.00 (reference)	
Yes	53 (16.0)	85 (6.1)	2.96 (2.05–4.27)	0.00	2.12 (1.42–3.18)	0.00

#### Factors associated with sexual intimate partner violence

In the regression model of sexual IPV, as shown in Table 6, compared with respondents with migration duration of 1–6 years, those with migration duration of 7–12 years (AOR 0.58, 95% CI: 0.40–0.84), migration duration of 13–20 years (AOR 0.62, 95% CI: 0.42–0.93) and migration duration of over 21 years (AOR 0.38, 95% CI: 0.21–0.71) were less likely to report sexual IPV. On the other hand, remarriage (AOR 2.73, 95% CI: 1.65–4.51), premarital sex (AOR 1.60, 95% CI: 1.18–2.14) and infidelity after marriage (AOR 2.54, 95% CI: 1.69–3.84) were identified as significant risk factors for sexual IPV.

**Table 6:** Factors associated with sexual IPV against married migrant workers

	sexual IPV		cOR (95% CI)	*p*	aOR (95% CI)	*p*
	Yes (*n* = 278)	No (*n* = 1466)				
	n (%)	n (%)				
Gender						
Men	125 (45.0)	748 (51.0)	1.00 (reference)		1.00 (reference)	
Women	153 (55.0)	718 (49.0)	1.3 (0.99–1.65)	0.06	1.31 (0.98–1.75)	0.07
Age group						
>37 years(first generation)	118 (42.4)	811 (55.3)	1.00 (reference)		1.00 (reference)	
≤37 years (second generation)	160 (57.6)	655 (44.7)	0.60 (0.46–0.77)	0.00	0.75 (0.53–1.05)	0.09
Education group						
Primary school or lower	25 (9.0)	122 (8.3)	1.00 (reference)		1.00 (reference)	
Junior high school	149 (53.6)	797 (54.4)	0.91 (0.57–1.45)	0.70	0.89 (0.55–1.46)	0.65
Senior high school	60 (21.6)	354 (24.1)	0.83 (0.50–1.38)	0.47	0. 66 (0.38–1.15)	0.15
College	44 (15.8)	193 (13.2)	1.11 (0.65–1.91)	0.70	0.76 (0.41–1.41)	0.39
Region of origin						
North	84 (30.2)	499 (34.0)	1.00 (reference)		1.00 (reference)	
South	194 (69.8)	967 (66.0)	1.19 (0.90–1.57)	0.22	1.34 (0.99–1.79)	0.06
Monthly income (RMB: yuan)						
≤2500	31 (11.2)	148 (10.1)	1.00 (reference)		1.00 (reference)	
2501–3500	135 (48.6)	711 (48.5)	0.91 (0.59–1.39)	0.65	1.04 (0.66–1.66)	0.86
>3500	112 (40.3)	607 (41.4)	0.88 (0.57–1.36)	0.57	0.99 (0.61–1.62)	0.99
Duration of migration (years)						
1–6	87 (31.3)	277 (18.9)	1.00 (reference)		1.00 (reference)	
7–12	83 (29.9)	456 (31.1)	0.58 (0.41–0.81)	0.00	0.58 (0.40–0.84)	0.00
13–20	88 (31.7)	543 (37.0)	0.52 (0.37–0.72)	0.00	0.62 (0.42–0.93)	0.02
≥21	20 (7.2)	190 (13.0)	0.34 (0.20–0.56)	0.00	0.38 (0.21–0.71)	0.00
Number of migratory cities						
1–2	153 (55.0)	901 (61.5)	1.00 (reference)		1.00 (reference)	
3–4	96 (34.5)	454 (31.0)	1.25 (0.94–1.65)	0.12	0.24 (1.20–0.89)	1.61
≥5	29 (10.4)	111 (7.6)	1.54 (0.99–2.40)	0.06	0.19 (1.41–0.85)	2.32
Marital status						
Frist marriage	241 (86.7)	1392 (95.0)	1.00 (reference)		1.00 (reference)	
Remarriage	37 (13.3)	74 (5.0)	2.89 (1.90–4.39)	0.00	2.73 (1.65–4.51)	0.00
Lifestyle with your spouse						
Living together	208 (76.2)	1217 (83.3)	1.00 (reference)		1.00 (reference)	
Living in separate places	65 (23.8)	244 (16.7)	1.56 (1.14–2.13)	0.01	1.17 (0.83–1.66)	0.36
Marital satisfaction						
Satisfied	206 (74.1)	1178 (80.4)	1.00 (reference)		1.00 (reference)	
Ok	63 (22.7)	258 (17.6)	1.40 (1.02–1.91	0.04	0.43 (0.82–1.62)	1.15
Dissatisfied	9 (3.2)	30 (2.0)	1.72 (0.80–3.67)	0.16	0.69 (0.34–2.02)	0.83
Premarital sex						
No	153 (55.0)	1018 (69.5)	1.00 (reference)		1.00 (reference)	
Yes	125 (45.0)	447 (30.5)	1.86 (1.43–2.42)	0.00	1.60 (1.19–2.14)	0.00
Marriage derailment						
No	224 (81.2)	1377 (94.1)	1.00 (reference)		1.00 (reference)	
Yes	52 (18.8)	86 (5.9)	3.72 (2.56–5.39)	0.00	2.54 (1.69–3.84)	0.00

## Discussion

This study estimated the prevalence, frequency and patterns of different types of IPV in a large sample of typical Chinese married rural-to-urban migrant workers, and identified the risk and protective factors for IPV at three levels: individual, relationship and societal level.

The first finding of the study is that as high as 45% of married rural-to-urban migrant workers experienced at least one act of physical, psychological or sexual IPV during the past 12 months. The incidence rate of IPV in this study is within the estimated range of IPV but is much higher than the findings of other Asian studies, including studies from other parts of China [31–33]. For instance, in the study conducted in western China, out of 1,502 rural women, 29.1% of subjects experienced IPV in last year and the prevalence of psychological IPV, physical IPV and sexual IPV was 26.6, 8.6, and 3.1% respectively among the investigated subjects [34]. This discrepancy could be related to various assessment scales used, different samples selected, different data analyses performed in these studies. In addition, up to 39.05% of subjects experienced at least one episode of psychological IPV, followed by 19.5% for physical IPV and 16.06% for sexual IPV. The present study, along with prior studies, indicates that psychological IPV is the most common type of domestic violence. Meanwhile, a growing body of research evidence suggests that psychological IPV does not always lead to physical IPV or sexual IPV directly, but physical IPV or sexual IPV in domestic relationships are nearly always preceded and accompanied by psychological IPV [1, 3, 8]. Thus, more attention should be paid for this common but neglected type of violence in the future studies.

The second important finding of the study is that among these domestic violence victims, over a half of them experienced multiple forms of violence from their intimate partners, especially three overlapping patterns of IPV. These findings are in line with most of previous studies [7, 14, 35, 36]. For example, in a current and influential study, Abeya S G, et al. surveyed 1,540 married/cohabited women and found that the overlap of the three forms of psychological, physical and sexual violence is the most commonly occurring form which accounted for 56.9% of the total violence [7]. It supports the view that most of domestic violence could be regarded as a combined pattern of different abuses rather than a relatively isolated incident [14].

The third important finding of the study is that some individual, relationship and societal factors are significantly associated with an increased or decreased likelihood of intimate partner violence against married rural-to-urban migrant workers.

At the individual level, the high level of education for the subjects was identified as a protective factor of IPV, especially physical IPV. Meanwhile, low level of education is the most consistent factor associated with both the perpetration and experiencing of overall IPV and physical violence across studies [37–40]. For example, those subjects with lower levels of education (primary or none) have a 2 to 5-fold risk of overall IPV compared to higher educated subjects [38–40]. This is justified as higher educational attainment enlarged an individual’s exposure and access to resources, decreased the acceptance of violence and improves socio-economic conditions, which could contribute to protect individuals from IPV.

Furthermore, compared to the first-generation rural-to-urban migrant workers, the second-generation rural-to-urban migrant workers are less likely to report current experience of overall IPV, especially psychological violence. This finding is inconsistent with results of previous studies which always identified young age to be a risk factor for intimate partner violence [7, 41, 42]. In China, compared with their fathers’ generation, the second-generation migrant workers have better education, higher level of urbanization and civilization. More importantly, most of them migrate from rural to urban areas in search of work as soon as they graduate from school or college; therefore the impact of traditional culture or ideas on them is limited. Thus, the new generation is less likely to report current experience of IPV than the older generation.

At the relationship level, low level of marital satisfaction was identified as a significant risk factor of IPV, especially psychological and physical IPV. In this study, respondents who reported dissatisfaction with their marriages were more than five times as likely to experience IPV as respondents who expressed satisfaction. Our findings are in line with previous research that individuals dissatisfied with their marriage are more likely to be involved in psychologically or physically abusive relationships than those individuals satisfied with their marriage [43, 44]. This association goes with Lewis and Fremouw’s explanation that violence and marital satisfaction are bidirectional, that there may be low satisfaction in the past which leads to violence or vice-versa [45].

In particular, premarital sex and extramarital affairs were two of the most consistent markers of IPV. On the one hand, premarital sex was considered a moral issue which was still taboo in traditional Chinese culture and typically unacceptable by the old generation. Hence, premarital sex usually enable an individual to suffer from poor self-image, low self esteem, feelings of embarrassment, humiliation and other risk factors contributing to violence in the family. On the other hand, extramarital affairs also are a strong risk factor of IPV. This is consistent with findings from other studies elsewhere [46–48]. Infidelity could be consider as one of the most serious threats that can jeopardize the marital relationship, especially the discovery of a spouse’s involvement in an extramarital affair is possibly one of the most stressful life events. Moreover, the sociocultural values of traditional culture in China may promote people to hold negative attitudes to extramarital affairs. Thus, extramarital affairs are often accompanied by various domestic violence and sometimes lead to the dissolution of a family.

At the societal level, duration of migration acted as a protective factor for IPV, especially physical IPV and sexual IPV. Specifically, long-term migrant workers are less likely to have experience of physical IPV and sexual IPV than short-term ones. It is possibly explained that migration from rural to urban areas is the process of urbanization which create great opportunities for migrant workers to get good education and improve their living standards. Furthermore, their traditional social norms supportive of violence may be transferred through urbanization processes. On the contrary, the increasing number of migratory cities appears to be a risk factor for being a victim of physical IPV. In this study, compared to respondents migrating to one or two cities, those migrating above five cities were about twice more likely to report physical IPV. It is clear that frequent moves could increase the likelihood of unstable living situations, and several studies have demonstrated that housing instability was a significant predictor of domestic violence [49, 50]. As such, our study confirmed that urbanization seems to decrease risk of IPV but placement instability could increase risk of IPV among the married rural-to-urban migrant workers.

We should acknowledge some limitations in our study. First and foremost, our study was based on cross-sectional design, which is not possible to get a valid cause-and-effect relation between intimate partner violence and the associated factors. To clarify the causality, we need longitudinal data or panel data for further research. Secondly, we only examine prevalence of intimate partner violence in past 12 months without including the prevalence of lifetime IPV, which may limit the generalizability of the findings to the target population. Third, information on experiences with IPV is very sensitive and private. In our study, data were self-reported in nature and respondents may be too embarrassed to reveal private details, which may be subject to reporting bias. Future studies should consider triangulating self-reports with clinical records, and health and social services records. Fourth, some relevant potential factors that are important to the IPV research were not included, such as participant’s drug use, alcohol, smoking, poverty, having children, being pregnant and different types of marriage. The investigation of these factors could be key directions for future research. Additionally, the use of the Conflict Tactics Scale to measure the psychological IPV, physical IPV and sexual IPV also limits the findings. Although the CTS scale has been widely used in a large number of studies on IPV, there have been several criticisms regarding this scale [51]. For instance, one of the criticisms is that the CTS only measure a small number of violent acts. It may be that more comprehensive research methods of IPV are needed to derive more reliable measures of the dimensions of IPV.

The present study has several strengths. First, the sample size was large enough to allow more reliable results with greater precision and power. In addition, interviewers and supervisors received systematic training and have past experiences of data collection; hence it was found a high response and prevalence rate of the study. Second, to our knowledge, this study is the first estimation of the prevalence, frequency and patterns of different types of IPV in a large sample of typical Chinese married rural-to-urban migrant workers. Thirdly, our study is one of the first studies to identify the risk and protective factors for IPV against migrant workers based on the ecological model (individual, relationship and societal level). It might offer a framework for understanding the associated factors that influence IPV, and can therefore provide key points for prevention and intervention of IPV among married rural-to-urban migrant workers.

## Conclusion

In conclusion, this study provides empirical evidence for the prevalence of different types of IPV against married migrant workers. Nearly half of married migrant workers experienced at least one incident of IPV during the past 12 months. Moreover psychological IPV was the most prevalent type of violence married rural-to-urban migrant workers experienced. Alarmingly, the joint occurrence of multiple forms of violence is the most commonly reported features of IPV, especially three overlapping patterns of IPV. Some individual (education and age), relationship (marital satisfaction, premarital sex and extramarital affairs) and social (duration of migration and number of migratory cities) factors of the respondents, were negatively or positively associated with IPV in the study area.

Given the massive number of married migrants in China, the domestic violence against them should receive urgent attention at all levels of societal organization including policymakers, stakeholders, professionals and other concerned body. Furthermore, despite limited resources, the screening, treatment, and intervention programs targeting IPV and its risk factors should be developed to identify, prevent, and reduce the occurrence and reoccurrence of IPV among the millions of migrant workers in China. Moreover, extensive and longitudinal research is needed to validate the current findings

## Additional files

Additional file 1: Data used in this paper. Description of data: data of socio-demographic information and intimate partner violence among migrant workers in eastern China. (XLS 1363 kb)
Additional file 2: A transcript of the questionnaire. Description of data: A socio-demographic questionnaire and a Chinese version of short form of the revised Conflict Tactics Scales (CTS2) (DOC 76 kb)

## Abbreviations

aOR: Adjusted odds ratios
CI: Confidence interval
cOR: Crude odds ratios
CTS: Conflict Tactics Scales
IPV: Intimate partner violence
