# Supplementary material for: Intimate partner violence against married rural-to-urban migrant workers in eastern China: prevalence, patterns, and associated factors
Source: BMC Public Health. 2016 Dec 7;16:1232. doi: 10.1186/s12889-016-3896-x (PMC5142315; doi:10.1186/s12889-016-3896-x)
Supplement: Additional file 2: — A transcript of the questionnaire. Description of data: A socio-demographic questionnaire and a Chinese version of short form of the revised Conflict Tactics Scales (CTS2) (DOC 76 kb) [file 12889_2016_3896_MOESM2_ESM.doc]

###### Questionnaire for Intimate Partner Violence among Married Migrant Workers

Hi, everyone：

Intimate partner violence is a growing public health problem in China. We are conducting a survey to gather information, about the patterns of intimate partner violence among married migrant workers; a scientifically clear understanding of the impact of domestic violence on married migrant workers will be used for making recommendations to all appropriate officials, on methods for reducing domestic violence among this important segment of China’s population. You have been randomly selected to participate in this study. Your participation will contribute to helping prevent domestic violence. Please carefully read and fill-in the questionnaire as accurately as you can remember. The information you provide will be strictly confidential, and will only be used for research purposes.

Thank you for your participation.

―― Department of Psychology, Wenzhou Medical University

**Instructions: please check“√” the number to indicate which response that best fits your experience.**

**A. Basic information**

|  | Gender： | （1）male | （2）female |  |
| --- | --- | --- | --- | --- |
| 2. | Age: |  |  |  |
| 3. | Duration of marriage | ________year | ________month |  |
| 4. | Education years | ________year |  |  |
| 5. | Region of origin | （1） north | （2）south |  |
| 6. | Monthly income | ________RMB |  |  |
| 7. | Duration of migration | ________year | ________month |  |
| 8. | Number of migratory cities | （1）1-2 | （2）3-4 | (3) ≥5 |
| 9. | Marital status | （1）Frist marriage | （2）Remarriage |  |
| 10. | Lifestyle with your spouse | （1）Living together | （2）Living in separate places |  |
| 11. | Marital satisfaction | （1）Satisfied | （2）Ok | (3) Dissatisfied |
| 12. | Premarital sex | （1）No | （2）Yes |  |
| 13. | Marriage derailment | （1）No | （2）Yes |  |
|  |  |  |  |  |

**B. Short form of the revised Conflict Tactics Scales (CTS2)**

**Instructions:** No matter how well a couple gets along, there are times when they disagree, get annoyed with the other person, want different things from each other, or just have spats or fights because they are in a bad mood, are tired or for some other reason. Couples also have many different ways of trying to settle their differences.This is a list of things that might happen when you have differences. Please mark how many times you did each to these things in the past year, and how many times your partner did them in the past year.

How often did this happen?

1 = Once in the past year 2 = Twice in the past year 3 = 3-5 times in the past year

4 = 6-10 times in the past year 5 = 11-20 times in the past year 6 = More than 20 times in the past year

| **1.** | I explained my side or suggested a compromise for a disagreement with my partner | 0 | 1 | 2 | 3 | 4 | 5 | 6 |
| --- | --- | --- | --- | --- | --- | --- | --- | --- |
| **2.** | My partner explained his or her side or suggested a compromise for a disagreement with me | 0 | 1 | 2 | 3 | 4 | 5 | 6 |
| **3.** | I insulted or swore or shouted or yelled at my partner | 0 | 1 | 2 | 3 | 4 | 5 | 6 |
| **4.** | My partner insulted or swore or shouted or yelled at me | 0 | 1 | 2 | 3 | 4 | 5 | 6 |
| **5.** | I had a sprain, bruise, or small cut, or felt pain the next day because of a fight with my partner | 0 | 1 | 2 | 3 | 4 | 5 | 6 |
| **6.** | My partner had a sprain, bruise, or small cut or felt pain the next day because of a fight with me | 0 | 1 | 2 | 3 | 4 | 5 | 6 |
| **7.** | I showed respect for, or showed that I cared about my partner’s feelings about an issue we disagreed on | 0 | 1 | 2 | 3 | 4 | 5 | 6 |
| **8.** | My partner showed respect for, or showed that he or she cared about my feeling about an issue we disagreed on | 0 | 1 | 2 | 3 | 4 | 5 | 6 |
| **9.** | I pushed, shoved, or slapped my partner | 0 | 1 | 2 | 3 | 4 | 5 | 6 |
| **10.** | My partner pushed, shoved, or slapped me | 0 | 1 | 2 | 3 | 4 | 5 | 6 |
| **11.** | I punched or kicked or beat-up my partner | 0 | 1 | 2 | 3 | 4 | 5 | 6 |
| **12.** | My partner punched or kicked or beat-me-up | 0 | 1 | 2 | 3 | 4 | 5 | 6 |
| **13.** | I destroyed something belonging to my partner or threatened to hit my partner | 0 | 1 | 2 | 3 | 4 | 5 | 6 |
| **14.** | My partner destroyed something belonging to me or threatened to hit me | 0 | 1 | 2 | 3 | 4 | 5 | 6 |
| **15.** | I went see a doctor (M.D.) or needed to see a doctor because of a fight with my partner | 0 | 1 | 2 | 3 | 4 | 5 | 6 |
| **16.** | My partner went to see a doctor (M.D.) or needed to see a doctor because of a fight with me | 0 | 1 | 2 | 3 | 4 | 5 | 6 |
| **17.** | I used force (like hitting, holding down, or using a weapon) to make my partner have sex | 0 | 1 | 2 | 3 | 4 | 5 | 6 |
| **18.** | My partner used force (like hitting, holding down, or using a weapon) to make me have sex | 0 | 1 | 2 | 3 | 4 | 5 | 6 |
| **19.** | I insisted on sex when my partner did not want to or insisted on sex without a condom (but did not use physical force) | 0 | 1 | 2 | 3 | 4 | 5 | 6 |
| **20.** | My partner insisted on sex when I did not want to or insisted on sex without a condom (but did not use physical force) | 0 | 1 | 2 | 3 | 4 | 5 | 6 |

**Please check whether you have filled in all items, thank you.**

If you agree, we wish to keep in touch with you.

phone_______________ email _______________

**Investigator______________**

**Checker______________**

**Survey date：______year _____month _____day**
